# Femtosecond Laser Fabricated Elastomeric Superhydrophobic Surface with Stretching-Enhanced Water Repellency

**DOI:** 10.1186/s11671-019-3140-6

**Published:** 2019-10-24

**Authors:** Huan Yang, Kaichen Xu, Changwen Xu, Dianyuan Fan, Yu Cao, Wei Xue, Jihong Pang

**Affiliations:** 1 Zhejiang Key Laboratory of Laser Processing Robot, College of Mechanical & Electrical Engineering, Wenzhou University, Wenzhou, 325035 China; 2 International Collaborative Laboratory of 2D Materials for Optoelectronics Science and Technology of Ministry of Education, College of Optoelectronic Engineering, Shenzhen University, Shenzhen, 518060 China; 3 Department of Electrical and Computer Engineering, National University of Singapore, 4 Engineering Drive 3, Singapore, 117576 Singapore; 4 Sino-German College of Intelligent Manufacturing, Shenzhen Technology University, Shenzhen, 518118 China

**Keywords:** Superhydrophobic surface, Silicone elastomer, Femtosecond laser, Wetting behavior, High stretchability

## Abstract

Highly stretchable and robust superhydrophobic surfaces have attracted tremendous interest due to their broad application prospects. In this work, silicone elastomers were chosen to fabricate superhydrophobic surfaces with femtosecond laser texturing method, and high stretchability and tunable adhesion of the superhydrophobic surfaces were demonstrated successfully. To our best knowledge, it is the first time flexible superhydrophobic surfaces with a bearable strain up to 400% are fabricated by simple laser ablation. The test also shows that the strain brings no decline of water repellency but an enhancement to the superhydrophobic surfaces. In addition, a stretching-induced transition from “petal” state to “lotus” state of the laser-textured surface was also demonstrated by non-loss transportation of liquid droplets. Our results manifest that femtosecond laser ablating silicone elastomer could be a promising way for fabricating superhydrophobic surface with distinct merits of high stretchability, tunable adhesion, robustness, and non-fluorination, which is potentially useful for microfluidics, biomedicine, and liquid repellent skin.

**Electronic supplementary material:**

The online version of this article (10.1186/s11671-019-3140-6) contains supplementary material, which is available to authorized users.

## Background

Artificial superhydrophobic surfaces play an important role in a variety of applications such as drag reduction [1], anti-biofouling [2], microfluidic manipulation [3], anti-icing [4–6], water collection [7], and wearable electronics [8]. For a promising superhydrophobic surface used in artificial skin and wearable electronics, high stretchability, durability, biological safety, and easy fabrication are highly desirable, so the proper selection of substrate materials and fabrication method is very crucial.

An approach to obtain high stretchability is fabricating superhydrophobic surfaces on elastic materials. For example, 3D wrinkle templates were usually used to transfer designed patterns onto elastomers with low surface energy [9]. However, the faithful replication of nanoscale structures remains a formidable challenge, as the elastomer curing in the nanoscale structure of template tends to break or deform during the peeling-off procedure. In recent studies, stretchable superhydrophobic surfaces fabricated by depositing hydrophobic micro/nanoparticles on pre-stretched elastic materials were reported [10, 11]; by this way, superhydrophobic surfaces could retain water-repellent property even at a stretching ratio of 500%. Nevertheless, the fabrication process is complicated and time-consuming, and the use of volatile organic compounds does not conform to the requirement of green manufacturing.

To generate hierarchical micro/nanostructures on rigid or flexible substrates, femtosecond laser processing/texturing is an easy and efficient approach, which has been employed in various applications [12–16]. With the property of cold processing, this technique has been proved to be an appropriate method to prepare low melting point flexible superhydrophobic surfaces [17–19]. The previous researches mainly focused on the texturing of polytetrafluoroethylene (PTFE) and polydimethylsiloxane (PDMS) [20, 21]. However, the tensile deformation of PTFE was irreversible [22], and the relatively low elastic modulus of PDMS limits the stretchability of its superhydrophobic surface to a strain below 100% [21].

Ecoflex is an ultra-soft flexible substrate, which can be stretched up to 500% and exhibits good mechanical compliance with human skin [23, 24]. Besides, this type of elastomer, being eco-friendly and harmless to the human body, has been widely used in wearable devices [25], so using it as a laser-textured substrate could be a solution to fabricate highly stretchable superhydrophobic surface. Hereby, in this study, highly stretchable, durable, and non-fluorinated superhydrophobic surfaces with controllable periodic structures were fabricated by femtosecond laser texturing of Ecoflex elastomers for the first time. With different laser processing parameters, micro/nanostructures can be regulated to determine the initial wetting behaviors of the silicone elastomers. The relationship of the wetting behaviors with respect to the strains was investigated. The flexible superhydrophobic surfaces with a bearable strain up to 400% are demonstrated. The mechanical stretching test also shows that the superhydrophobic surfaces feature stretching-enhanced water repellency. Meanwhile, the relevant mechanism was discussed.

## Methods and Experiment

### Materials

The flexible rubber (Ecoflex 00-20) was purchased from Smooth-On, Inc., USA.

### Preparation of Silicone Elastomers

**Fig. 1:**
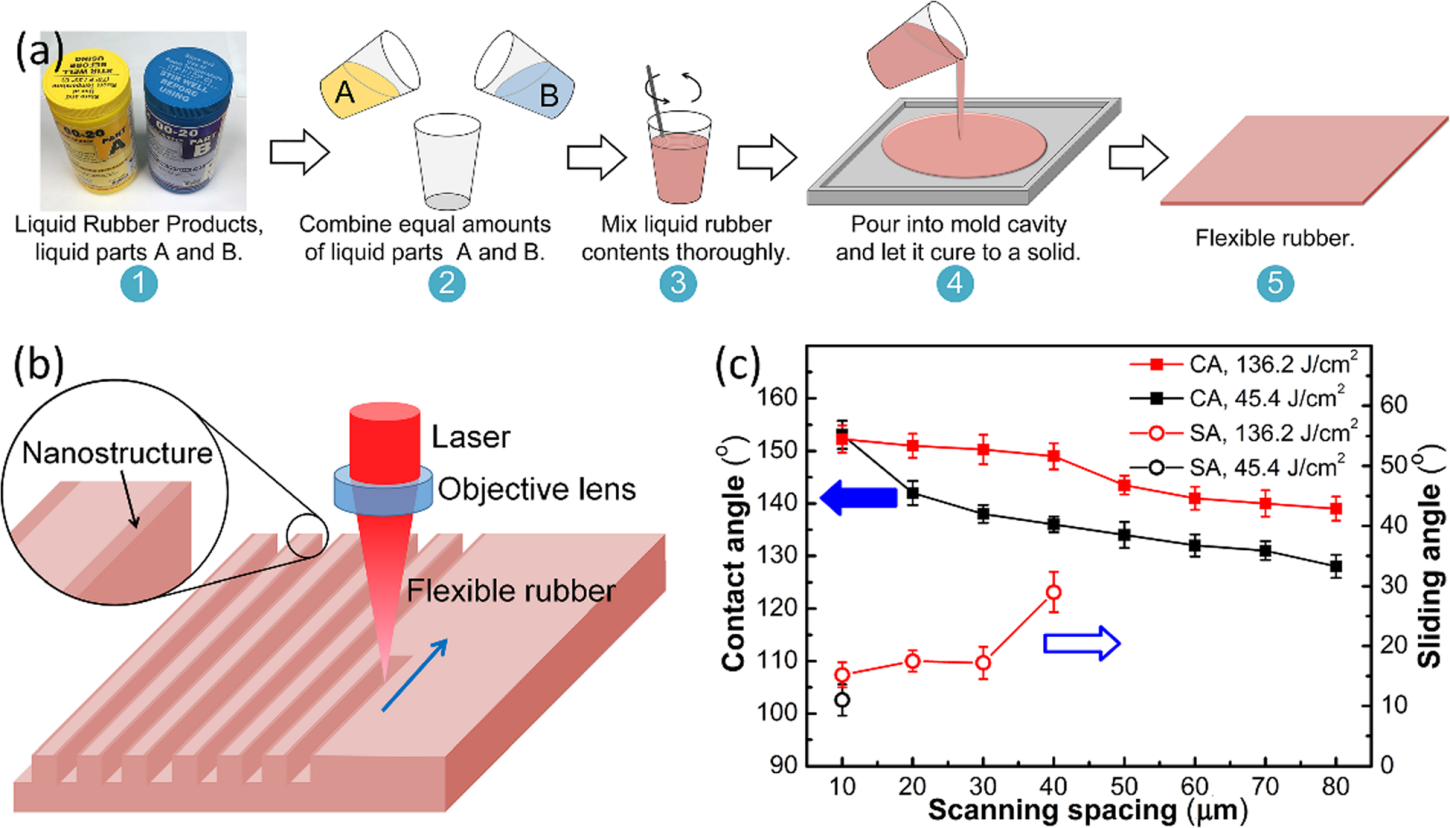
**a** Fabrication process of solid Ecoflex rubber. **b** Schematic device configuration and fabrication process. **c** Effect of laser processing parameters on CAs and SAs

As shown in Fig. 1a, the flexible rubber with a thickness of 2 mm was prepared by mixing liquid parts A and B with a volume ratio of 1:1 and allowed to cure completely in a mold for 12 h at room temperature [23].

### Fabrication of Elastomeric Superhydrophobic Surfaces

The micro/nanoscale hierarchical structures on silicone elastomer were fabricated by femtosecond laser ablation through a line-by-line scanning in the air (Fig. 1b). The silicone rubber was mounted on a nanotechnology stage (XY-Tripod-Theta 6 Axis System, Alio Industries) and then irradiated by a Ti:sapphire femtosecond laser system (LIBRA, Conherent Inc., CA, USA) with a pulse width of 100 fs at 1 kHz pulse repetition frequency and a central wavelength of 800 nm. The Gaussian laser beam was focused by an objective lens (× 10, Nikon, Japan) with a numerical aperture (NA) of 0.24, and the laser scanning speed was fixed at 2 mm/s. The processing parameters for achieving the superhydrophobic surface were optimized by changing the scanning spacing and the laser fluence.

### Characterization

The surface morphology of the laser-textured silicone elastomer was characterized using a scanning electron microscope (SEM, JEOL JSM-7001F) and a laser scanning confocal microscope (OLYMPUS, OSL4100). Energy-dispersive X-ray spectroscopy (EDS) measurements were made to evaluate the chemical changes on the laser-ablated surface. The contact angle (CA) and sliding angle (SA) were measured by a contact angle meter (SEO PHOENIX).

## Results and Discussion

### Structure and Superhydrophobic Properties

The wetting modes of diverse artificial water-repellent surfaces are based on the surface morphology inspired by biomineral materials [26]. Low adhesion (LA) superhydrophobic surfaces mimicking lotus leaves are endowed with low sliding angles below 10° [27], and high adhesion (HA) superhydrophobic surfaces derived from rose petals [28] have distinct characteristics that water drops cannot slide from the surface at any titled angle. In this paper, the two kinds of surface morphology were both fabricated by laser texturing the elastomer with different processing parameters [29].

**Fig. 2:**
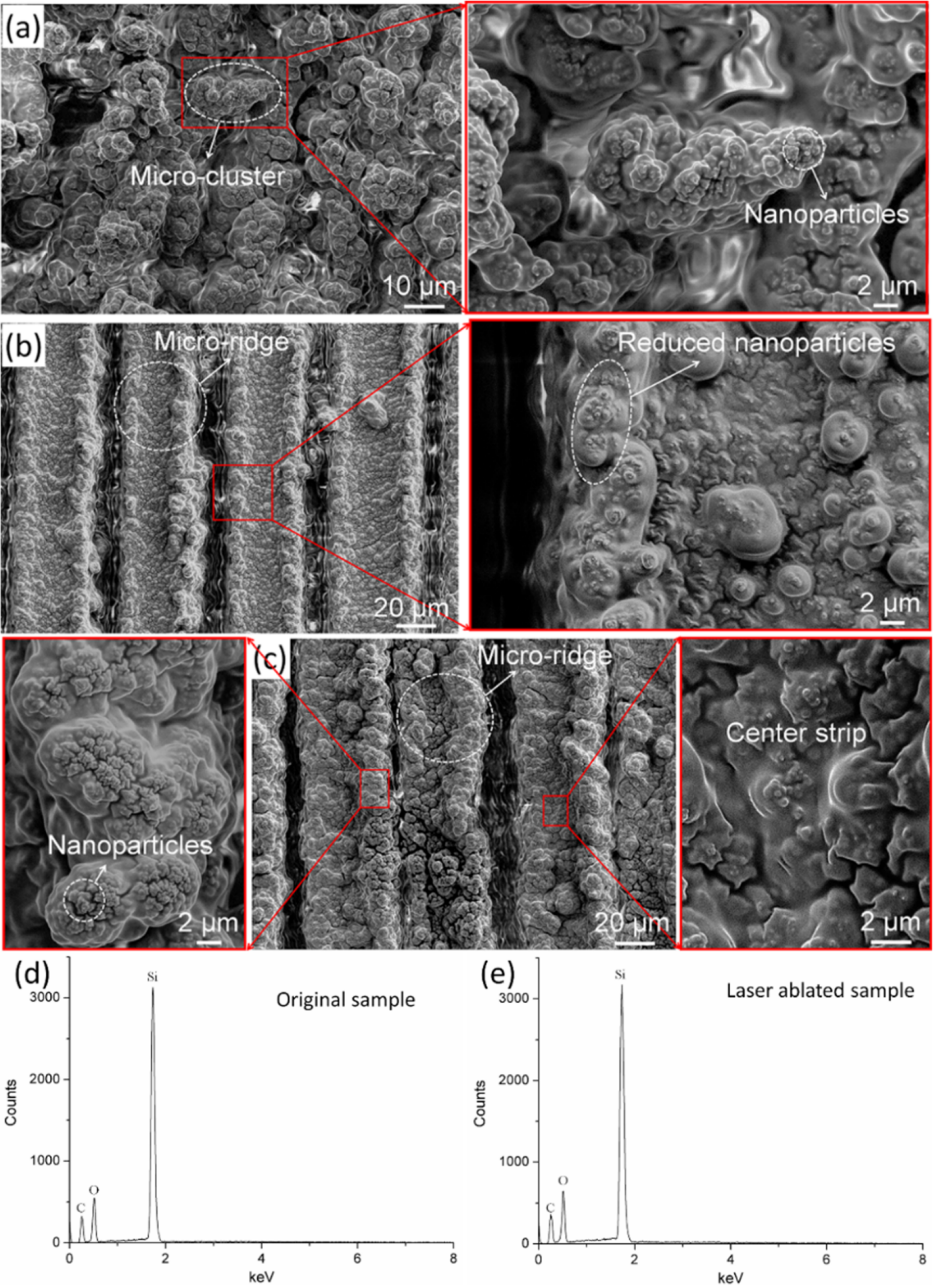
SEM images of the femtosecond laser-induced rough microstructures with different laser fluences and scanning spacings. **a** 45.4 J/cm^2^, 10 μm. **b** 45.4 J/cm^2^, 50 μm. **c** 136.2 J/cm^2^, 50 μm. EDS spectrum record for original sample (**d**) and laser-ablated sample (**e**)

Figures 1c and 2a–c show the wetting properties and surface morphologies of the laser-textured silicone elastomers. The missing SA data in Fig. 1c represents the HA superhydrophobic surface with an SA of 180°. As shown in Fig. 2, the laser-ablated surface possesses a typical micro/nanoscale hierarchical structure, where the cluster-like (Fig. 2a) and groove-shaped (Fig. 2b, c) microscale patterns are achieved by the removal of material. Besides, these microscale structures are covered by the nanoparticles with the size of 100–200 nm, which are induced by the rapid cooling of the ejected liquid melt in the localized melt region [30]. Moreover, the EDS spectrum test shows that the chemical changes induced by femtosecond laser patterning of the elastomer surface are not significant (Fig. 2d, e), only a slight increase in the oxygen content. When the laser fluence is 45.4 J/cm^2^ and the scanning spacing is 10 μm, the laser-ablated surface shows excellent superhydrophobicity where the CA is 153.1° and SA is 11°. As the scanning spacing increases, the CA decreases gradually (Fig. 1c), and the droplet on the surface became motionless even if the sample is tilted by 180°. When the scanning spacing increases to 80 μm, the CA decreases to 128°. When the laser fluence is 136.2 J/cm^2^ and the scanning spacing is 80 μm, the ablated surface can still obtain a CA over 140° (CA = 141.5°).

According to the surface morphology illustrated in Fig. 2, the surface texturing can be divided into two parts. One locates at the edges of the microridges, exhibiting a microscale raised structure with rich nanoparticles. Nanostructures have been proven to be a key factor for superhydrophobic properties [31]; the air layer trapped in this type of structure not only prevents droplet from penetrating into the groove vacancy, but also enables a small solid/liquid contact area which causes low adhesion. However, the center part of the microridge is flat comparing to the edge and lacks of nanostructure (Fig. 2c), which results in complete contact and high adhesion in solid/liquid interfaces. With fixed laser fluence, the width of the center flat part on the microridge is decided by the scanning spacing, so the total adhesion force increases as the scanning spacing increases. Hence, considering the processing efficiency and the sample performance, the laser fluence was fixed at 136.2 J/cm^2^, and the spacings of 30 μm and 50 μm were chosen to prepare the LA and HA superhydrophobic surfaces, respectively.

### Strain-Modulated Structures and Wettability

Because the superhydrophobic surface was fabricated in the line-by-line scanning way, the relationship of CA and SA with respect to the strain was investigated by pulling the tensile superhydrophobic surface in the directions perpendicular (⊥) and parallel (∥) to the laser scanning orientation. The strain value (*ε*) is defined by the equation *ε* = (*L* − *L*_0_)/*L*_0_, where *L* and *L*_0_ are the lengths of the elastomer at the stretched state and the initial state, respectively.

**Fig. 3:**
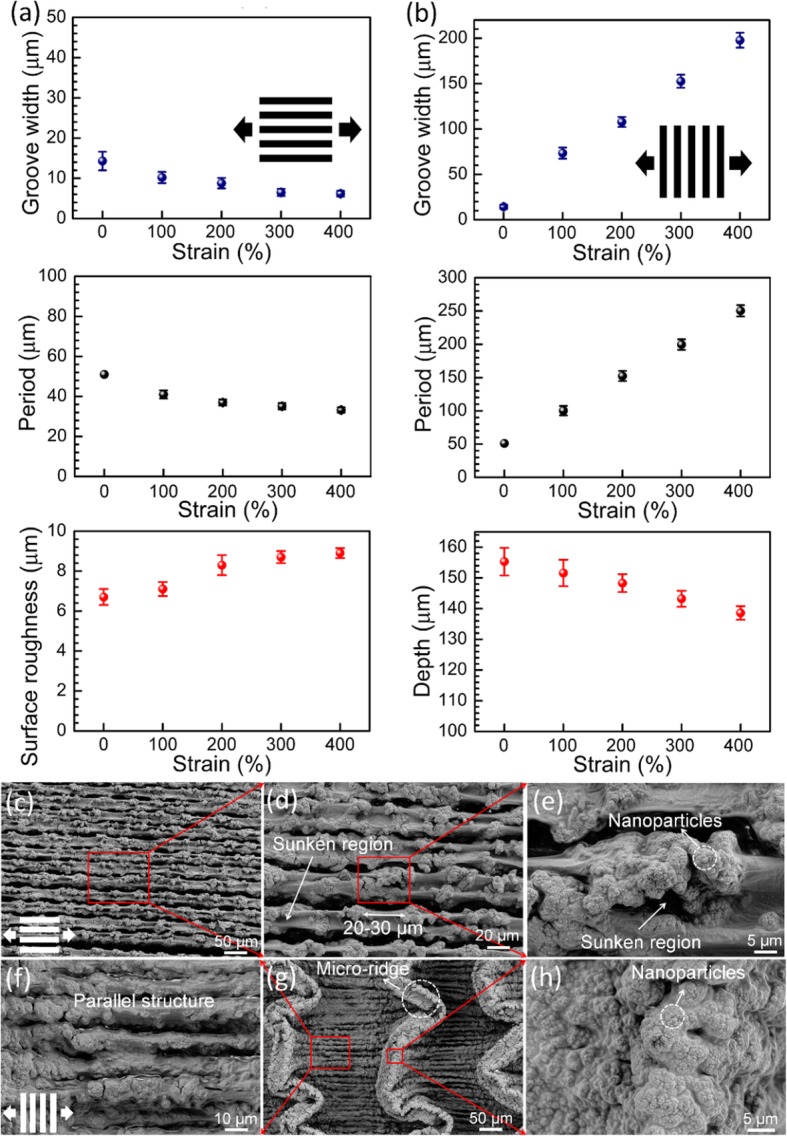
Structural parameters of the HA superhydrophobic elastomer stretched at 0–400% strain in the parallel direction (**a**) and perpendicular direction (**b**). Surface morphologies of the HA superhydrophobic elastomer stretched at the strain of 400% in the parallel (**c**–**e**) and perpendicular (**f**–**h**) directions

Figure 3 a and b show the structural parameters of the stretched superhydrophobic elastomers as a function of the parallel and perpendicular strain values. When the laser-textured specimen is pulled in the parallel direction, the parallel strain compresses the grating and results in a decreased period and groove width (Fig. 3a, c). Meanwhile, the center strip of the microridge becomes folded and is covered by the surrounding micro/nanoscale structures (Fig. 3e). The elongated microridge forms a new hierarchical structure with a period of 20–30 μm at the strain of 400% (Fig. 3d), which enriches and diversifies the surface structure. On the other hand, the exertion of the perpendicular stretching leads to linear growth of period as well as groove width and a little decrease of groove depth (Fig. 3b), but the width and surface morphology of the microridges keep almost unchanged (Fig. 3f–h). A parallel structure with the period of about 10 μm is formed at the bottom of the microgrooves (Fig. 3f).

**Fig. 4:**
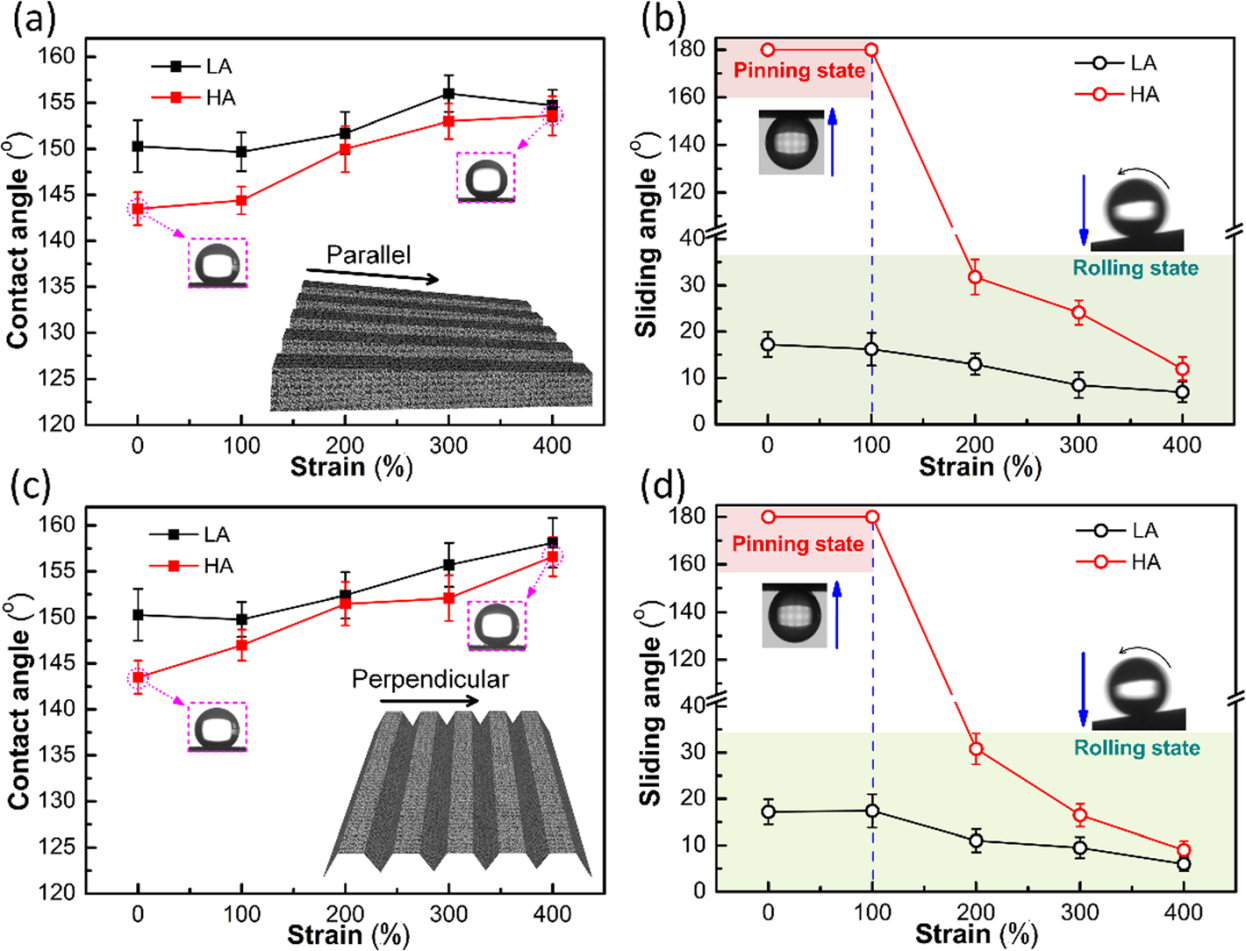
CAs (**a**) and SAs (**b**) of the superhydrophobic elastomers at different parallel strain values. CAs (**c**) and SAs (**d**) of the surface at different perpendicular strain values

Figure 4 shows the effects of parallel strain and perpendicular strain on the CAs and SAs of laser-ablated superhydrophobic surfaces. As the tensile stress increases, for both LA and HA superhydrophobic surfaces, an obvious improvement of superhydrophobic behavior is demonstrated. This result is in contrast to the previous reports [21, 32], in which the mechanical stretching led to the decline of water repellency. Especially for the HA superhydrophobic surface, when the strain is 100%, the CA is 144.4°, and the water droplet is stuck on the rough surface even in an upside-down state (Fig. 4b), which is called as “pinning state.” As the strain increases to 200%, the CA rises to 150°. Meanwhile, the water droplet slides off with a 43° tilt angle, which reveals that the wetting state is changed to “rolling state.” When the strain reaches 400%, the HA superhydrophobic surface obtains the most excellent superhydrophobicity with 153.6° CA and 12° SA. When the specimen is pulled along the perpendicular direction, as shown in Fig. 4c, d, for both the LA and HA superhydrophobic surfaces, the variation curves of CAs and SAs are both similar to the results obtained in the parallel tensile test (Fig. 4a, b), and the increase of CAs is more linear. The state change of the HA superhydrophobic surface occurs as well with a strain of 200%, and as the strain increases to 400%, the HA superhydrophobic surface could obtain a maximum CA of 156.6° and a minimum SA of 9°.

### Mechanism of Stretching-Enhanced Water Repellence

**Fig. 5:**
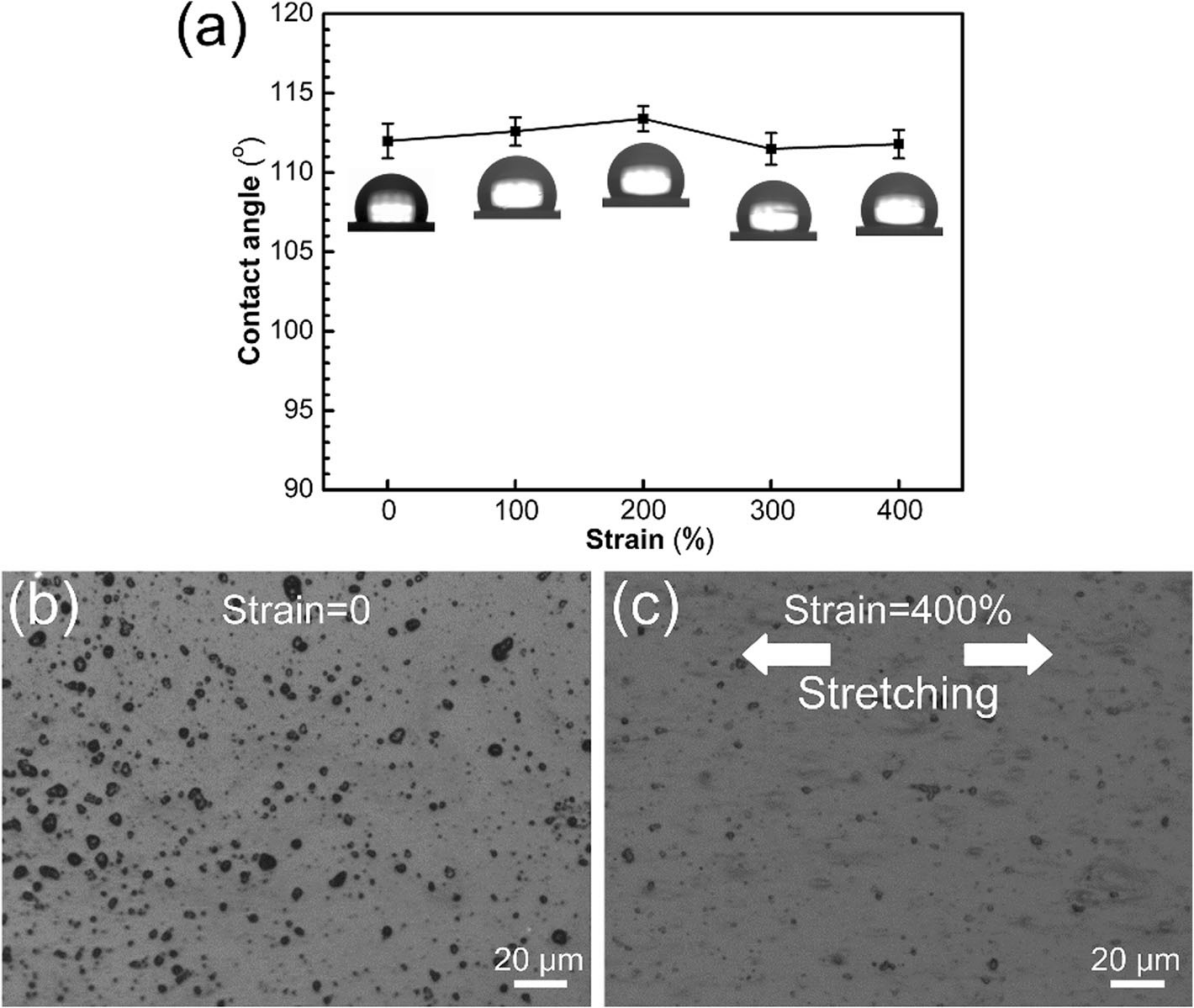
**a** CAs of the original elastomer at different strain values, and microscope images of the original elastomer with the strain of (**b**) 0 and (**c**) 400%

The above results demonstrate that enhanced superhydrophobicity could be obtained after the superhydrophobic elastomer was stretched with a strain above 100%, along the direction either perpendicular or parallel to the laser scanning orientation. For the starting sample which is not processed by femtosecond laser, the surface morphology and superhydrophobicity remain the same after being pulled up with a strain of 400% (Fig. 5). And no chemical transformation occurs during the stretching process, so the enhanced wetting behavior should be attributed to the variation of surface morphology.

In this paper, to understand the increase of superhydrophobicity of the stretched silicone elastomer, a combined state is employed to explain the wettability of the superhydrophobic elastomer [33]. The whole solid-liquid interaction of the superhydrophobic elastomer can be described by the Cassie-Baxter model, but the interaction in the wetted central region on the microridge is in the Wenzel state. According to the Cassie-Baxter model in the air [34], the CA (*θ*_C_) in the air/liquid/solid system can be expressed as the following equations:

1 $$ \cos {\theta}_{\mathrm{C}}={f}_{\mathrm{S}}\cos {\theta}_{\mathrm{S}}-{f}_{\mathrm{A}} $$

where *f*_S_ and *f*_A_ are the fractions of the solid/water interface and the air/water interface (*f*_S_ + *f*_A_ = 1), respectively, and *θ*_S_ is the ideal CA on the smooth silicone elastomer (for Ecoflex 00-20, *θ*_S_ = 112°, Fig. 5). The CA in the wetted central region which complied with the Wenzel model can be presented as follows:

2 $$ \cos {\theta}_{\mathrm{W}}=r\cos {\theta}_{\mathrm{S}} $$

where *θ*_W_ is the CA in the Wenzel model, and *r* is the surface roughness factor defined as the ratio of the actual surface area to the projected area. By ignoring the air pockets trapped in the nanostructures, the CA (*θ*) in the combined state can be expressed with the following approximate equations [35]:

3 $$ \cos \theta ={f}_{\mathrm{S}}\left(r\cos {\theta}_{\mathrm{S}}+1\right)-1 $$

According to Eq. 2, in the Wenzel model, *r*cos*θ*_S_ is a value between − 1 and 1, so the value of (*r* cos *θ*_S_ + 1) in Eq. 3 must be a positive value.

**Fig. 6:**
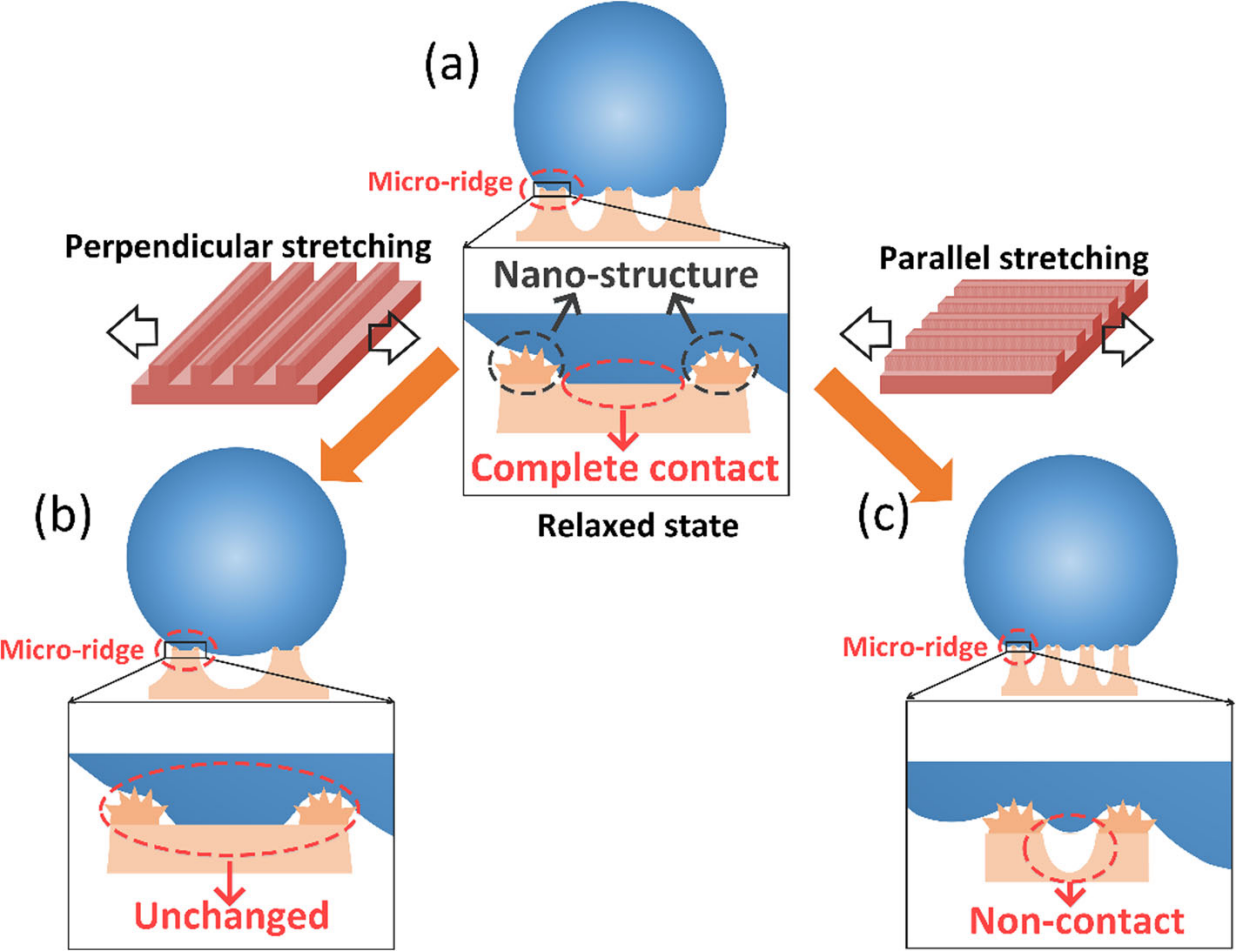
Cross-section schematic illustration of (**a**) the relaxed sample, and the samples stretched in the (**b**) perpendicular direction and (c) parallel direction

Figure 6 illustrates the cross-sectional schematic diagrams of droplets on the superhydrophobic surfaces with different tensile states. For the relaxed superhydrophobic surface (Fig. 6a), when the laser-textured specimen is pulled in the perpendicular direction, the solid/liquid contact area of the single microridge almost remains unchanged (Figs. 3g, h and 6b), and it means that the *r* in Eq. 3 is kept as a constant, but the fraction of the whole solid/water interface (*f*_S_) continues to decrease, which results in the increase of *θ*. Moreover, the increased CA and groove width (Fig. 3b and 6a) both decrease the number of microridges in contact with the droplet, which leads to the decrease of the total adhesion force. For the tilted HA superhydrophobic surface, if the adhesion force drops to a value smaller than the tangential in gravity, the droplet slides off from the superhydrophobic surface. For the parallel stretching, the surface area of the microridge and the width of the grooves are both decreased (Fig. 6c), indicating that the fraction of the solid/water interface (*f*_S_) is almost kept consistent. However, thanks to the sunken region at the center of microridges (Fig. 3e and 6c) and the emerging hierarchical structure along the stretching direction (Fig. 3d), the surface roughness factor (*r*) increases, which leads to the increase of *θ*. The significantly reduced solid/liquid contact area of the single microridge can also induce a weakened adhesion force, which contributes to the transformation from “pinning state” to “rolling state” for the HA superhydrophobic surface.

### Durability

**Fig. 7:**
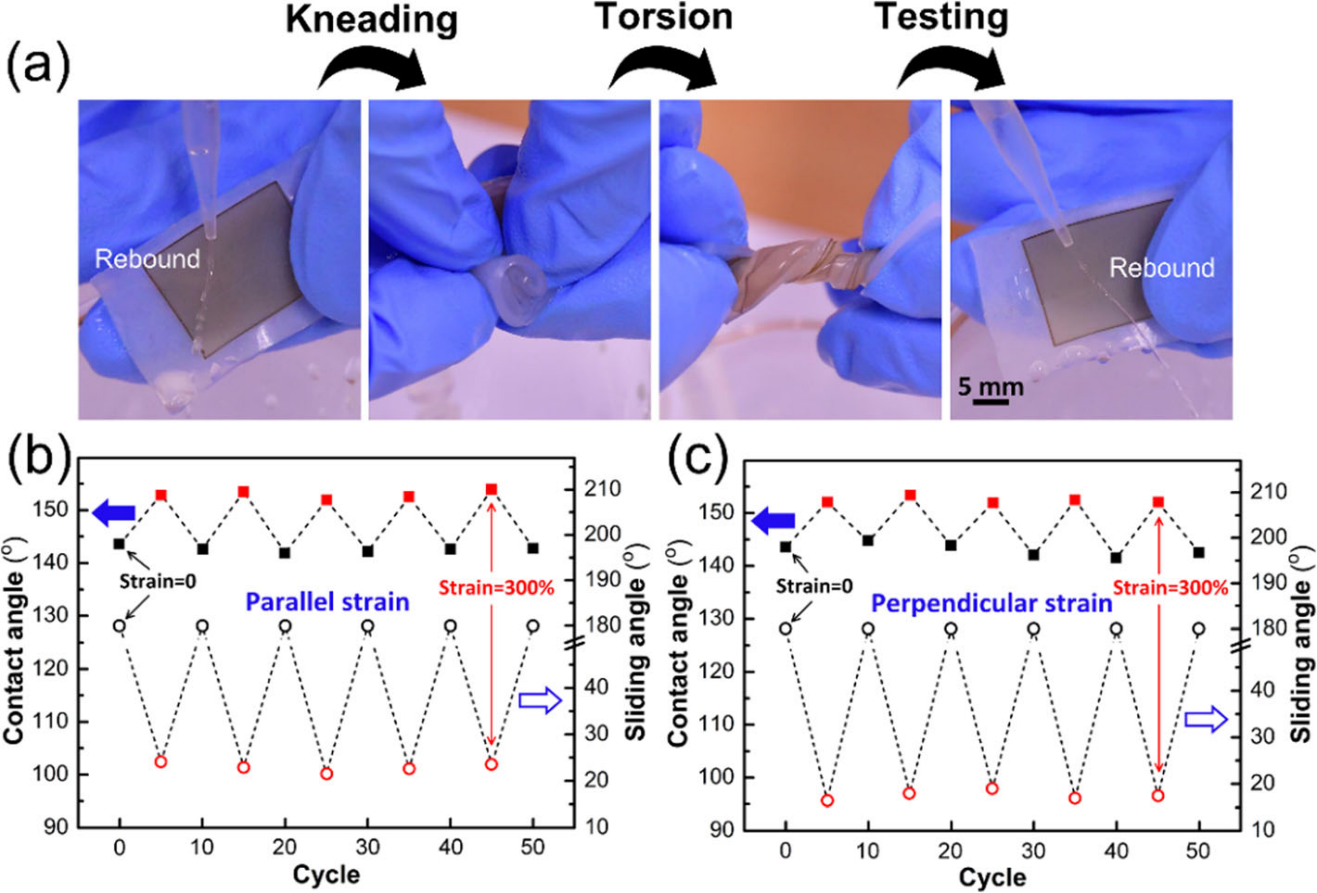
**a** Processes of kneading and torsion and cyclic tests of stretching-relaxing conducted in the (**b**) parallel and (**c**) perpendicular directions for the HA superhydrophobic elastomer

The durability of the highly stretchable superhydrophobic surface is an important parameter in practical applications. Figure 7a shows how to test the durability. The superhydrophobic elastomer is rolled up, kneaded and distorted again and again, and then measured. For the LA superhydrophobic elastomer, the elastomer can still completely bounce the water jetting to the surface after 50 loops of distortion, which indicates that the rough surface possesses satisfactory stability. For the HA superhydrophobic elastomer, cyclic tests of stretching-relaxing at a 300% strain are conducted both in the parallel (Fig. 7b) and perpendicular (Fig. 7c) directions, and the superhydrophobic properties in relaxed and stretched state are tested at 10 cycle intervals. During the 50 cycles of stretching-relaxing, the HA superhydrophobic elastomer reveals high reversibility and repeatability for the dynamic transformation from “pinning state” to “rolling state.”

### Droplet Transportation

**Fig. 8:**
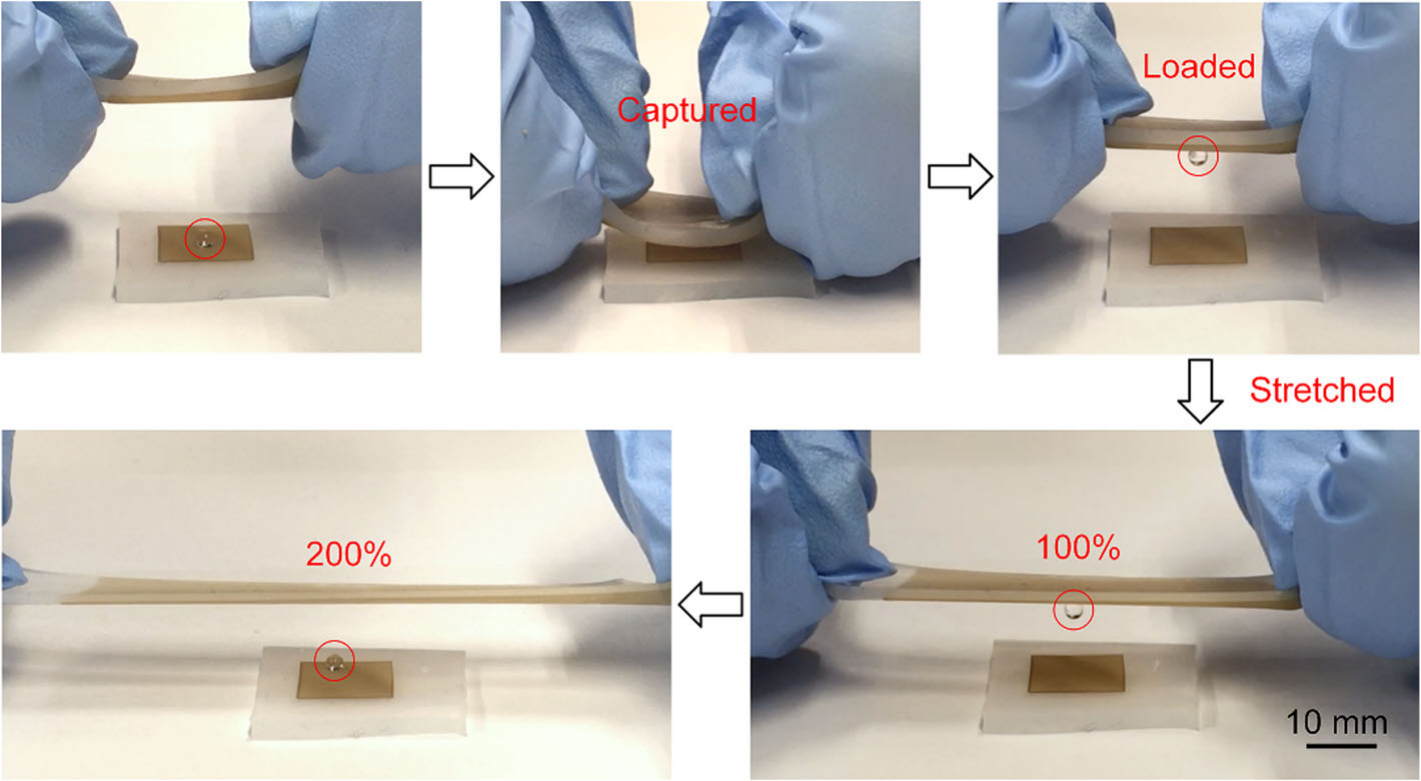
Demonstration of the lossless droplet transfer using the stretchable HA superhydrophobic elastomer

When simple mechanical stretching and relaxing is applied alternately to the HA superhydrophobic surface, a reversible and repeatable transition from “pinning state” to “rolling state” can be readily realized, so this type of surface can be employed in effective and safe transportation of tiny droplets, especially for expensive and rare liquid samples. An illustration of the transportation process is shown in Fig. 8. A water droplet with a volume of 5 μL is initially placed on an LA superhydrophobic surface, and an HA superhydrophobic surface is approaching and making contact to the droplet from the above. Due to the strong adhesion force of the HA surface, the droplet can be captured, lifted, and transported without loss. By stretching the elastomer, the adhesion force between the solid/liquid interface reduces until the gravity on the droplet wins out, and the droplet is thus released. A video (Additional file 1: Video S1) is also provided to demonstrate the whole process. This unsophisticated mechanism can be easily integrated into an automated robotic device which is of great significance for lab-on-chip applications. Besides, with the rapid development of laser technology, high-frequency femtosecond lasers with power exceeding 100 W can be produced [36], and the new galvanometer technology can achieve a scanning speed above 100 m/s [37]. So based on the high-power femtosecond laser and high-speed galvanometer, the large-scale industrial of laser-fabricated stretchable superhydrophobic surfaces is possible.

## Conclusions

Robust fluorine-free superhydrophobic surfaces capable of sustaining ultra-high strain (400%) have been successfully fabricated on a commercial silicone elastomer by femtosecond laser texturing for the first time. Based on the controllable micro/nanoscale structures determined by laser processing parameters, the initial wetting performances can be flexibly managed. Furthermore, by stretching the surface, the superhydrophobicity is not weakened but enhanced to a certain extent, no matter which direction is the stretching force applied in. With an HA superhydrophobic surface, liquid droplets could be captured and released through stretching and releasing cycles. The surface water repellency property is well retained after multiple cycles of kneading and torsion, which indicates a good endurance and exceptional value of applicability. The highly stretchable surface with manageable superhydrophobicity presented in this work is highly promising for biomedicine, microfluidics, and intelligent wearable devices.

## Additional file

Additional file 1: **Video S1.** A water droplet can be captured and released by a single superhydrophobic elastomer. (MP4 1281 kb)

## Data Availability

The datasets generated and/or analyzed during the current study are available from the corresponding author on request.

## Abbreviations

CA: Contact angle
HA: High adhesion
LA: Low adhesion
PDMS: Polydimethylsiloxane
PTFE: Polytetrafluoroethylene
SA: Sliding angle
